# Ascending Aortic Thrombus After SARS-CoV-2 Infection

**DOI:** 10.7759/cureus.22496

**Published:** 2022-02-22

**Authors:** Julian Yet Kwong Horman, Noreen Petrash, Jennifer Kraschnewski, Puja Patel

**Affiliations:** 1 Internal Medicine, Penn State Health Milton S. Hershey Medical Center, Hershey, USA; 2 Pediatrics, Penn State Health Children's Hospital, Hershey, USA

**Keywords:** renal infarction, aortic thrombus, covid-related hypercoagulability, covid-19, sars-cov-2

## Abstract

A 46-year-old male with a history of SARS-CoV-2 infection one month ago presented to the hospital due to acute onset left flank pain. He was found to have an acute left renal embolic infarction from a large 15mm ascending aortic thrombus, which may have formed due to a transient hypercoagulable state from his recent SARS-CoV-2 infection along with tobacco use. He was medically managed with anticoagulation for six months. Subsequent imaging after three months of therapeutic anticoagulation showed complete resolution of the ascending aortic thrombus.

## Introduction

SARS-CoV-2 has many known complications, including hypercoagulability. Unfortunately, due to the relative novelty of this infection, optimal therapies for thromboembolic events secondary to SARs-CoV-2 infection are not well established.

Hypercoagulability is a complication that has been widely documented with SARS-CoV-2 infections. The prothrombotic state seen with SARS-CoV-2 is likely multifactorial, although the exact mechanism is not fully understood. Currently, the hypercoagulable state is believed to be due to an interplay between the inflammatory cascade along with endothelial dysfunction that ultimately leads to fibrin clot formation caused by the SARS-CoV-2 virus [1]. Studies have shown that SARS-CoV-2 is able to infect vascular endothelial cells, which may partially explain the widespread endothelial damage that is seen in SARS-CoV-2 infections [2]. Furthermore, SARS-CoV-2 infection causes an inflammatory cascade, which includes interleukins, that causes vascular endothelium to transition to an activated state. The activated endothelium is then able to promote inflammation by recruiting inflammatory cells, such as neutrophils, that can alter the thrombotic potential of the endothelial surface [3].

SARS-CoV-2 has been shown to cause thrombosis, involving numerous body systems as well as both the arterial and venous systems. There are cases of SARS-CoV-2 causing acute limb ischemia, mesenteric ischemia, myocardial infarction, acute cerebrovascular accidents, and deep vein thrombosis, although this is certainly not an exhaustive list [4]. Cases of aortic thrombosis due to SARS-CoV-2 are rare, and there are only a few documented cases in the literature. Of the published cases of aortic thrombi due to SARS-CoV-2, most are seen in patients with severe disease [5].

## Case presentation

**Figure 1:**
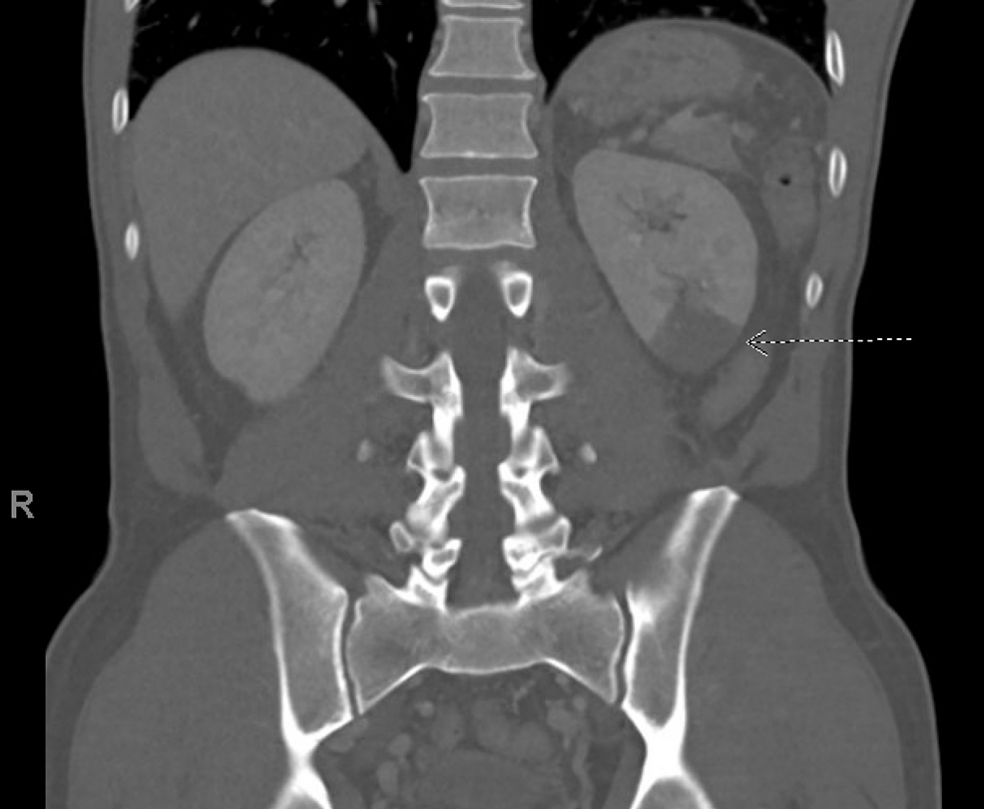
CT of the abdomen with the white arrow pointing at the left inferior pole hypodensity concerning for infarction

The patient is a 46-year-old male with a history of diverticulosis and tobacco use disorder who presented to the hospital due to one day of acute onset lower back pain and abdominal pain. The pain developed about an hour after eating dinner and continued to worsen throughout the night with development of nausea and frequent bowel movements without diarrhea. The pain did not improve with oxycodone 5mg with acetaminophen 325mg. The patient denied dysuria, fevers, chest pain, and shortness of breath. About one month prior to presentation, he tested positive for SARS-CoV-2 via nasal PCR testing. His symptoms were mild, and he completed a 14-day quarantine at home, only requiring symptomatic treatment. A CT of the abdomen and pelvis with contrast was performed due to the patient’s abdominal and back pain, which showed a new wedge-shaped hypodensity in the inferior pole of the left kidney concerning for infarction (Figure 1).

**Figure 2:**
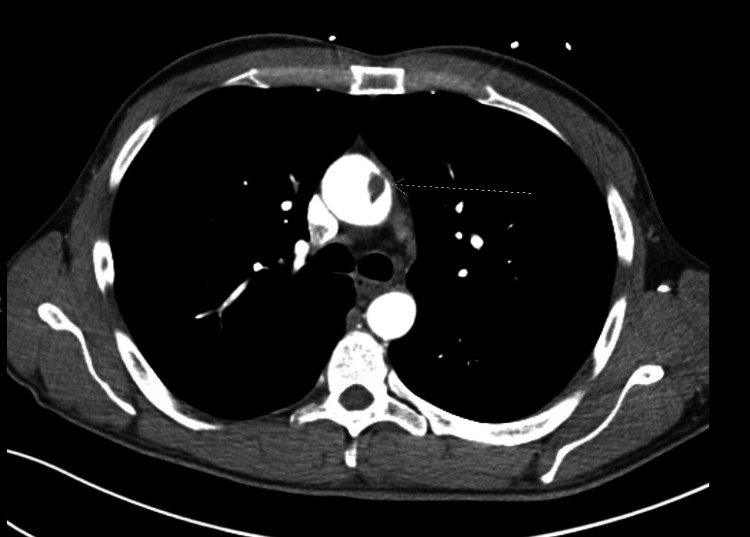
CT angiography of the ascending aorta with the white arrowing pointing at the large 15mm free-floating thrombus

**Figure 3:**
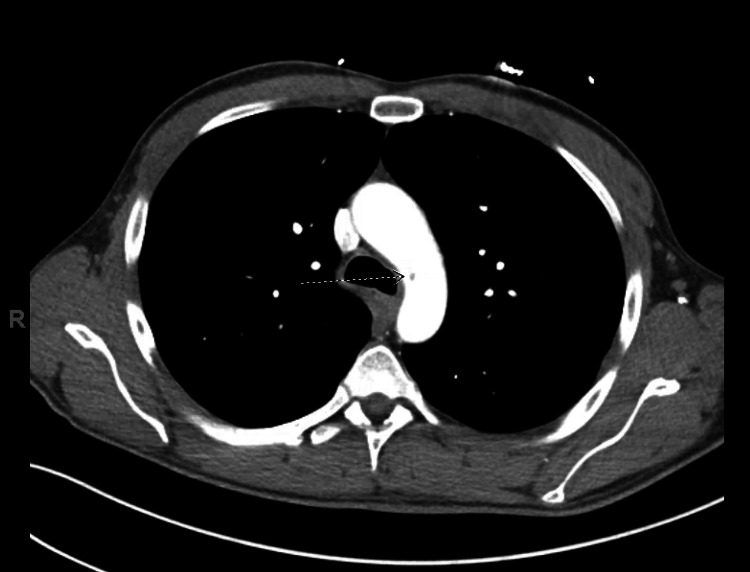
CT angiography of the aortic arch with the white arrow pointing at a small thrombus

Given the finding of a renal infarction, further imaging with CT angiogram of the chest and abdomen was performed to search for embolic sources. CT angiogram of the chest revealed a free-floating 15mm thrombus within the ascending aorta (Figure 2) and a small free-floating thrombus in the aortic arch (Figure 3).

A transthoracic echocardiogram was performed, which was unremarkable, without any extension of the thrombus into the heart or any aortic valve interruption. Cardiac surgery was consulted due to the CT angiogram findings. Due to the size and location of the thrombus, it was determined that the risks of surgical intervention were too great and that medical management would be a safer approach. Therefore, the patient was started on a therapeutic heparin infusion. A hypercoagulable workup was performed, including protein C, protein S, antithrombin III, prothrombin, factor V Leiden, lupus anticoagulant/antiphospholipid antibodies, homocysteine, cardiolipin, B-2 glycoprotein, and lipoprotein A, all of which were normal. Due to the negative hypercoagulable workup and previous SARS-CoV-2 diagnosis, the most likely etiology of the thrombus was a transient hypercoagulable state post-SARS-CoV-2 infection. He was continued on the heparin drip until discharge and was subsequently transitioned to apixaban 10mg twice daily for seven days and then 5mg twice daily for six months. The patient was instructed to refrain from heaving lifting until resolution of the thrombus. After three months of continuous anticoagulation therapy, a follow-up CT angiogram of the chest was performed, which showed complete resolution of the aortic thrombi; therefore, the patient’s activity restrictions were eased. He was followed by hematology, and after six months the apixaban was discontinued and he was transitioned to aspirin 81mg daily. He was followed closely by his primary care physician during the six-month follow-up period, and he did not report any complications due to the thrombus or anticoagulation during this period.

## Discussion

Thrombotic complications occur in an estimated 0.8-31% of patients with SARS-CoV-2 infections [6,7]. While severe disease is associated with higher incidence of thrombotic complications, mild disease also carries an increased risk of thrombosis [8,9]. This patient, who had a previous mild SARS-CoV-2 infection, still experienced a significant thrombotic event. This highlights the increased morbidity associated with SARS-CoV-2 infection, even with mild disease.

This patient presented with an ascending aortic thrombus about one month after his initial SARS-CoV-2 infection. Much of the current data regarding thrombus risk due to SARS-CoV-2 is during an acute infection. Data regarding the risk of thrombosis after SARS-CoV-2 infection are limited but there does appear to be a continued risk of thrombosis for 90 days post-SARS-CoV-2 infection [10]. There are case reports of patients having laboratory evidence of prothrombotic changes up to four months after SARS-CoV-2 infection [11]. While the risk of thrombosis after SARS-CoV-2 infection does appear to continue after the initial infection, the role of thromboprophylaxis after SARS-CoV-2 infection remains controversial. The European Society of Cardiology recommends prolonged thromboprophylaxis if the International Medical Prevention Registry on Venous Thromboembolism (IMPROVE-VTE) score is greater than or equal to 4. Patients with D-dimer that is two times greater than the reference value may benefit from thromboprophylaxis. They also recommend thromboprophylaxis in patients with risk factors for thromboembolism and in patients with ongoing oncological processes [12].

Ultimately, an interdisciplinary team settled on six months of continued anticoagulation and imaging at three months, which showed resolution of the thrombus. Current venous thromboembolism treatment guidelines from the American Society of Hematology recommend three to six months of anticoagulation for initial provoked venous thromboembolism, which was the basis for the treatment duration in this case [13]. However, these guidelines were adopted in this case since they are specific to venous thromboemboli, as there are no clear guidelines regarding anticoagulation duration for aortic thrombosis. One case report of a patient with an aortic thrombus after SARS-CoV-2 infection assessed for resolution of the thrombus after only two weeks of therapy with a repeat CT scan [14]. In addition, a case report of an aortic thrombus secondary to SARS-CoV-2 infection was treated with coumadin for six months with an international normalized ratio goal of 2.5 to 3.5 [15]. These cases all highlight the fact that the optimal duration of therapy is not well understood currently nor is the optimal anticoagulant. Direct oral anticoagulants have been shown to adequately treat aortic thrombi due to SARS-CoV-2 infection, but there have not been any head-to-head studies comparing outcomes between the different direct oral anticoagulants or vitamin K antagonists. Furthermore, current therapy guidelines state that a duration of three to six months appears to adequately treat aortic thrombi; however, the optimal duration of therapy is not well understood.

## Conclusions

SARS-CoV-2 predisposes patients to both venous and arterial thromboembolism, including ascending aortic thrombus. The current guidelines for anticoagulation recommend three to six months of continuous anticoagulation for first-time, provoked thromboemboli, but the optimal duration for anticoagulation of SARS-CoV-2 induced thromboemboli is not well understood. Further studies are needed to further elucidate the optimal duration of anticoagulation for SARS-CoV-2 induced thromboemboli. Ascending aortic thrombus can be a complication of both severe and mild SARS-CoV-2 infection, and it can even be seen in patients with resolved SARS-CoV-2 infections. While it is a relatively rare finding, it can carry significant morbidity and mortality and therefore should be a consideration in patients presenting with unexplained embolic events.

## References

[1] Subramanian SV, Kumar A (2021). Increases in COVID-19 are unrelated to levels of vaccination across 68 countries and 2947 counties in the United States. Eur J Epidemiol.

[2] Nägele MP, Haubner B, Tanner FC, Ruschitzka F, Flammer AJ (2020). Endothelial dysfunction in COVID-19: current findings and therapeutic implications. Atherosclerosis.

[3] Siddiqi HK, Libby P, Ridker PM (2021). COVID-19 - a vascular disease. Trends Cardiovasc Med.

[4] Avila J, Long B, Holladay D, Gottlieb M (2021). Thrombotic complications of COVID-19. Am J Emerg Med.

[5] de Carranza M, Salazar DE, Troya J (2021). Aortic thrombus in patients with severe COVID-19: review of three cases. J Thromb Thrombolysis.

[6] Malas MB, Naazie IN, Elsayed N, Mathlouthi A, Marmor R, Clary B (2020). Thromboembolism risk of COVID-19 is high and associated with a higher risk of mortality: a systematic review and meta-analysis. EClinicalMedicine.

[7] Roubinian NH, Dusendang JR, Mark DG, Vinson DR, Liu VX, Schmittdiel JA, Pai AP (2021). Incidence of 30-day venous thromboembolism in adults tested for SARS-CoV-2 infection in an integrated health care system in Northern California. JAMA Intern Med.

[8] Douillet D, Riou J, Penaloza A (2021). Risk of symptomatic venous thromboembolism in mild and moderate COVID-19: a comparison of two prospective European cohorts. Thromb Res.

[9] Clavijo MM, Vicente Reparaz ML, Ruiz JI (2021). Mild COVID-19 illness as a risk factor for venous thromboembolism. Cureus.

[10] Li P, Zhao W, Kaatz S, Latack K, Schultz L, Poisson L (2021). Factors associated with risk of postdischarge thrombosis in patients with COVID-19. JAMA Netw Open.

[11] von Meijenfeldt FA, Havervall S, Adelmeijer J (2021). Sustained prothrombotic changes in COVID-19 patients 4 months after hospital discharge. Blood Adv.

[12] Begic E, Naser N, Begic N (2021). Hypercoagulability in COVID-19 and post-COVID patients - characteristics and current treatment guideline. Eur Soc Cardiol.

[13] Ortel TL, Neumann I, Ageno W (2020). American Society of Hematology 2020 guidelines for management of venous thromboembolism: treatment of deep vein thrombosis and pulmonary embolism. Blood Adv.

[14] Mullan C, Powierza C, Miller PE, Geirsson A, Vallabhajosyula P, Assi R (2020). Spontaneous coronavirus disease 2019 (COVID-19)-associated luminal aortic thrombus. J Thorac Cardiovasc Surg.

[15] Douedi S, Alshami A, Odak M, Patel S (2021). COVID-19-induced aortic thrombosis. Chest.

